# Kidney Injury Biomarkers in Leptospirosis

**DOI:** 10.1590/0037-8682-0260-2022

**Published:** 2023-01-23

**Authors:** Pablo Uribe-Restrepo, Claudia Munoz-Zanzi, Piedad Agudelo-Flórez

**Affiliations:** 1 Universidad CES, Escuela de Graduados, Medellín, Antioquia, Colombia.; 2 University of Minnesota, School of Public Health, Minneapolis, Minnesota, United States of America.

**Keywords:** Leptospira, Leptospirosis, Biomarkers, Acute kidney injury, Kidney disease

## Abstract

Leptospirosis is a zoonotic infection with a global distribution, though it has a greater impact on marginalized rural agricultural and urban communities in developing countries. Kidney injury, which can lead to severe and lethal infections, is the most frequent complication associated with leptospirosis. Novel biomarkers are being studied as tools for assessing kidney injury in different pathological processes to improve early detection. This review aimed to gather information on the use of novel kidney biomarkers for human leptospirosis. A search of the literature was carried out in September 2021 using the parameters “((kidney) OR (renal) OR (chronic kidney disease) OR (acute kidney injury)) AND ((biomarker) OR (marker)) AND ((*Leptospira*) OR (leptospirosis))”. The review identified 11 original studies that evaluated the performance of 15 kidney biomarkers related to leptospirosis. Assessment of the evidence for biomarker utility was limited because of the small number of studies and sample sizes. Although some biomarkers were associated with kidney disease, no specific biomarker appeared to be ready for clinical practice, and more research in this field is necessary.

## INTRODUCTION

*Leptospira* is a genus of highly mobile spirochaete bacteria comprising both pathogenic and saprophytic species. The interaction between a pathogenic species and susceptible host can lead to leptospirosis. Human leptospirosis is a reemerging zoonosis with a global distribution, presenting an average of 1.03 million cases and 58,900 deaths each year^1^. Leptospirosis ranges from mild or asymptomatic to deadly. The kidney is one of the main targets of pathogenesis in *Leptospira*-host interaction, and during acute disease, renal impairment is common, and bacterial persistence within the kidney leads to fibrosis^2^. 

Acute kidney injury (AKI) is one of the most frequent complications of leptospirosis, with a reported occurrence varying between 10% and 88% of leptospirosis cases^3^. Chronic kidney disease (CKD) may result after a resolved episode of acute leptospirosis, secondary to damage associated with the acute phase and/or due to the persistence of *Leptospira* in the kidney^4^. Herath *et al.* identified CKD after leptospirosis-related AKI in 9% of the patients in a two-year prospective study^5^. 

Chronic kidney disease of unknown etiology (CKDu) has emerged as a cause of CKD in agricultural communities in warm tropical regions, with toxins, infectious agents, and heat-related kidney stress proposed as probable causes^6^. Among the different explanations for CKDu, some authors suggest *Leptospira* could play a role in the etiology, as the bacteria have kidney tropism and regional hotspots are located in areas in which both diseases overlap^7^ ^,^ ^8^.

Kidney damage results in diminished function and is classified according to the time of establishment and the degree of function reduction^9^. Damage that is established in a matter of hours or a few days results in AKI, which constitutes a medical emergency and can lead to fatal illness^10^. CKD is established over time and implies more than three months of altered function that generally involves structural changes within the kidney. Individuals with CKD may not present symptoms until the advanced stages of the disease^11^.

Assessing kidney damage by quantifying renal function using traditional biomarkers such as serum creatinine (SCr), the derived estimated glomerular filtration rate (eGFR), or blood urea nitrogen (BUN) is an insensitive approach, as significant kidney injury may have already occurred prior to any change in these measures of kidney function. In contrast, the levels of kidney injury biomarkers that are produced by the pathological mechanism of injury may increase before kidney function is altered^12^. Consequently, kidney injury biomarkers have emerged as a promising tool in the diagnosis, prognosis, and monitoring of kidney disease, such as for the detection of AKI, prediction of poor outcomes in an emergency department setting^13^, and identification of patients at risk for kidney disease progression post-hospitalization^14^.

In the context of human leptospirosis, several kidney injury biomarkers have been studied for their potential use as tools for the early detection of kidney injury and to monitor patients after acute disease to identify those who might develop CKD^9^ ^,^ ^12^ ^,^ ^15^. Early detection improves the prognosis of CKD by allowing the early implementation of proper interventions^16^. This review aimed to gather available information on the measurement of kidney injury biomarkers in leptospirosis, underlining the performance of each biomarker in AKI and CKD.

## METHODS

**FIGURE 1:**
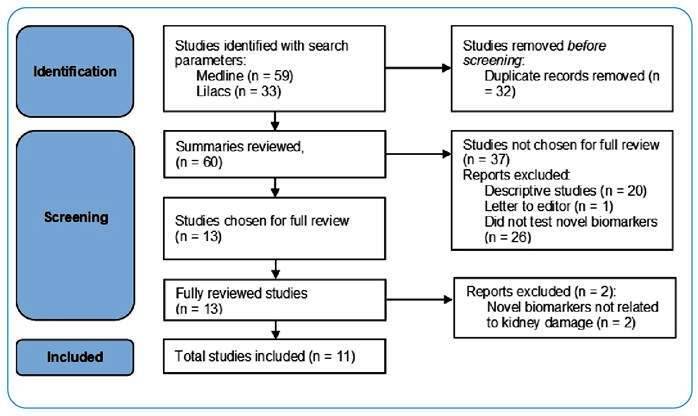
Flow diagram of study literature search and selection.

The MEDLINE and LILACS databases were searched in September 2021 using the following search parameters: “((kidney) OR (renal) OR (chronic kidney disease) OR (acute kidney injury)) AND ((biomarker) OR (marker)) AND ((*Leptospira*) OR (leptospirosis))”. The search did not have a year restriction for publication date. Summaries of the resulting articles were reviewed to apply the inclusion and exclusion criteria, as shown in Figure 1. The inclusion criteria were as follows: the article could be in any language, reporting original data on blood or urine biomarkers associated with kidney injury, and be from humans or animals with leptospirosis determined by positive laboratory tests. Studies with a wide description of biomarker profiles but did not evaluate specific biomarker performance were excluded, as well as studies that only evaluated classic kidney function biomarkers, such as SCr, BUN, or urine albumin. Data extracted from the selected articles included biomarkers measured, year of publication, country, sample size, human or animal study, biospecimen used, diagnosis of leptospirosis, and type of study design used to recruit participants. The Preferred Reporting Items for Systematic Reviews and Meta-Analyses guidelines for scoping reviews were adapted to report the results of the search.

## RESULTS

The initial search yielded 60 articles, including 59 from MEDLINE and 33 from LILACS, with 32 shared results. All studies were published between 1982 and 2021, with a median publication year of 2015. Fifty-eight studies were conducted in English, one in Danish, and one in Spanish. After applying the inclusion and exclusion criteria (Figure 1), there were 11 articles available for data extraction (Table 1). All the studies were written in English and published between 2014 and 2021. Fifteen biomarkers were evaluated in the selected studies. Four biomarkers (neutrophil gelatinase-associated lipocalin (NGAL), N-acetyl-β-D-glucosaminidase (NAG), kidney injury molecule 1 (KIM-1), and monocyte chemoattractant protein-1 (MCP-1)) had been evaluated in more than one study. The studies included cross-sectional, cohort, and comparative designs; two studies were conducted in dogs, and the overall sample size was 135 (Table 1). 

**TABLE 1:** Studies on kidney injury biomarkers associated to *Leptospira.*

Biomarker (fluid)	Study type (country)	Model (n)	Leptospira diagnosis	Results	Authors
ROS (blood)	Cohort (Brazil)	Human (n = 12)	MAT ≥ 1:800, seroconversion, or ELISA	Neither ROS or GSH showed significant differences in leptospirosis patients with or without AKI	Araújo *et al.* 2014^40^
GSH (blood)					
Angpt-2 (blood)	Retrospective cohort (Germany)	Human (n = 13)	Confirmed cases registered	Blood Angpt-2, ADMA and SDMA showed significantly elevated levels in leptospirosis cases with AKI than in leptospirosis cases without AKI	Lukasz *et al.* 2014^47^
ADMA (blood)					
SDMA (blood)					
NGAL (blood)	Cohort (Brazil)	Human (n = 77)	MAT ≥ 1:800 or seroconversion	Blood NGAL showed no correlation with creatinine levels	Libório *et al.* 2015^22^
Syndecan-1 (blood)				Both Syndecan-1 and ICAM-1 correlated with the presence of leptospirosis, Syndecam-1 associated with AKI	
ICAM-1 (blood)					
KIM-1/Cr (urine)	Population survey and cohort (Taiwan)	Human (n = 88)	MAT	Urinary KIM1/Cr ratio is higher in cases with MAT titer above 400. Urinary and Blood NGAL and Blood MCP-1 showed no difference between groups.	Yang *et al.* 2015^21^
NGAL (urine)					
NGAL (blood)					
MCP-1 (blood)					
NGAL (urine)	Cohort (Thailand)	Human (n = 206)	Mat ≥ 1:100, culture, or PCR	Both blood and urine NGAL levels were related with AKI in patients with and without leptospirosis	Srisawat *et al.* 2015^15^
NGAL (blood)					
Defensin α1 (urine)	Comparative study (Philippines)	Human (n = 135)	MAT, ELISA, PCR	Levels of Defensin α1, NGAL and NAG were adjusted using creatine, with significant differences in the levels of NGAL and NAG	Chagan-Yasutan *et al.* 2016^23^
NGAL (urine)					
NAG (urine)					
IL18 (urine)	Comparative study with nested cohort (Nicaragua)	Human (n = 489)	Mat ≥ 1:100 or PCR	In cohort subjects seropositive after a period with increased risk of exposure had higher levels of NGAL and NAG. All three biomarkers were higher in seropositive subjects.	Riefkohl *et al.* 2017^20^
NGAL (urine)					
NAG (urine)					
NAG (urine)	Comparative study (Philippines)	Human (n = 142)	MAT, ELISA, PCR	Blood levels of FL-OPN and FL-Gal-9 were higher in leptospirosis patients in urine samples NAG/Cr levels had the greatest ability to discriminate leptospirosis patients from healthy controls	Chagan-Yasutan *et al.* 2020^24^
FL-OPN (urine)					
FL-Gal-9 (blood)					
FL-Gal-9 (urine)					
NGAL (urine)	Cohort (Italy)	Dog (n = 206)	Mat ≥ 1:800, MAT seroconversion, or PCR	Urinary NGAL did not differ in AKI dogs with leptospirosis and AKI dogs without leptospirosis	Zamagni *et al.* 2020^19^
KIM-1 (urine)	Comparative study (Brazil)	Dog (n = 30)	PCR, culture, or Mat ≥ 1:800	There were statistically significant differences in the levels or urinary KIM-1	Dias *et al.* 2021^29^
KIM-1 (urine)	Comparative study (Sri Lanka)	Human (n = 170)	Positive MAT, PCR, or culture	Levels of both blood and urine KIM-1 and MCP-1 were higher in leptospirosis patients with AKI	Nisansala *et al. 2021* ^28^
KIM-1 (blood)					
MCP-1 (urine)					
MCP-1 (blood)					

**ROS:** Reactive oxygen species; **GSH:** Antioxidant reduced glutathione; **Angpt-2:** Angiopoietin-2; **ADMA:** Asymmetric Dimethylamines; **SDMA:** Symmetric dimethylarginines; **NGAL:** Neutrophil Gelatinase Associated Lipocalin; **ICAM-1:** Intercellular Adhesion Molecule 1; **KIM-1:** Kidney injury molecule 1; **Cr:** Creatinine; **MCP-1:** Monocyte chemoattractant protein-1; **NAG:** Urinary N-acetyl-β-D-glucosaminidase; **IL18:** Interleukin-18; **FL-OPN:** full-length osteopontin; **FL-Gal-9:** full length Galectin 9.

### Neutrophil Gelatinase-Associated Lipocalin (NGAL)

NGAL was included in six studies, two of which used NGAL as a reference point for the performance of other biomarkers. NGAL, also known as lipocalin 2, is a protein belonging to the lipocalin family and is present in neutrophil granules and epithelial cells from different organs, including the kidney^17^ ^,^ ^18^. Srisawat *et al.* carried out a multicenter cohort study in Thailand and found that both serum and urinary levels of NGAL were strongly linked with AKI in leptospirosis patients^15^. A cross-sectional study in dogs by Zamagni *et al.* found no differences in NGAL levels in leptospirosis-induced AKI when compared to other AKI causes^19^. Based on a cross-sectional serological study of 489 participants combined with a nested cohort of 282, Riefkohl *et al*. reported that NGAL levels were significantly elevated among *Leptospira* seropositive workers relative to seronegative workers, but the effect was reduced after further adjustment by job category^20^. Similar findings were reported by Yang *et al*. in a cohort study of 88 participants in which there was no association between NGAL and *Leptospira* microagglutination (MAT) levels^21^. Other studies have included NGAL as a reference point to test other biomarkers of leptospirosis-associated AKI. A cohort study by Libório *et al.* compared biomarker levels among 46 leptospirosis cases and 31 healthy controls and found a correlation between NGAL and SCr levels. In this study, NGAL was used as an acute injury marker to study the performance of syndecam-1 and intercellular adhesion molecule 1 (ICAM-1)^22^. Chagan-Yasutan *et al.* carried out a similar retrospective study with 143 participants in which NGAL was used as a reference point to evaluate the performance of defensin α1. In this study, NGAL levels were significantly different between leptospirosis cases and control groups^23^.

### Urinary N-acetyl-β-D-glucosaminidase (NAG)

NAG was the second most studied kidney injury biomarker and was included in three studies. Similar to NGAL, NAG was used as a reference point to test other biomarkers^20^ ^,^ ^23^ ^,^ ^24^. NAG is a high-molecular-weight lysosomal enzyme present in various organs, with a high concentration in the proximal renal tubule^25^. Chagan-Yasutan *et al.* published a retrospective study in which urinary NAG was used as a kidney injury biomarker in emergency department patients with leptospirosis in Thailand in 2016, and a second retrospective study was published in 2020. In both studies, NAG was strongly associated with AKI and had specificity for leptospirosis^23^ ^,^ ^24^. In a study by Riefkohl *et al.*, urinary NAG levels were higher in *Leptospira* seropositive participants than in seronegative participants; however, this was not statistically significant after further model adjustment^20^. 

### Kidney injury molecule 1 (KIM-1)

KIM-1, also known as cell immunoglobulin mucin domain 1 (TIM-1), is a type 1 transmembrane protein that increases rapidly during proximal tubule injury and has been linked to the pathogenesis of renal fibrosis^26^ ^,^ ^27^. This biomarker has been reported in three studies. Yang *et al.* evaluated its performance in a cohort study with a two-year follow-up in which leptospirosis MAT antibody titers >1:400 were associated with deterioration in renal function. Patients with high MAT titers also had higher urine KIM-1 and creatinine levels^21^. A cross-sectional study by Nisansala *et al.* found elevated urine KIM-1 levels in patients with leptospirosis related AKI^28^. Another cross-sectional study on naturally infected dogs by Dias *et al.* showed an association between high levels of urine KIM-1 and AKI as well as evidence that KIM-1 is an early marker of kidney injury^29^.

### Monocyte chemoattractant protein-1 (MCP-1)

MCP-1 is a protein from the chemokine family that plays a role in the migration and filtration of monocytes^30^. It is a mediator in acute ischemic and toxic kidney injury as well as a marker of kidney fibrosis^31^ ^,^ ^32^. Yang *et al.* assessed its capacity as a biomarker in a cohort study and found no link between MCP-1 levels and decreased kidney function^21^. In contrast, a cross-sectional study by Nisansala *et al.* found a link between elevated MCP-1 levels and leptospirosis-related AKI^28^.

### Interleukin-18 (IL-18)

Interleukin-18 (IL-18) is a member of the interleukin-1 family and has regulatory effects on innate and acquired immunity^33^. This biomarker was reported in a single study. In the combined cross-sectional and cohort study by Riefkohl *et al.*, investigators reported higher IL-18 levels in *Leptospira* seropositive participants than in seronegative participants; however, this was not statistically significant^20^.

### Syndecan-1

Syndecan-1 is a major cell surface heparan sulfate proteoglycan in epithelial cells that acts as an adhesion target for bacteria in many infections, and is an endothelial damage biomarker^34^. A cross-sectional study by Libório *et al.* with 77 participants showed that blood levels of syndecan-1 were higher in leptospirosis patients with AKI than in patients without AKI^22^.

### Defensin α1

Defensin α1 is an antimicrobial peptide that was effective against *Leptospira* in an *in vitro* model^35^ ^,^ ^36^. High levels of defensin α1 have been reported in inflammation leading to diabetic nephropathy^37^. Chagan-Yasutan *et al.* measured defensin α1 in a case-control study that evaluated AKI in 112 patients with leptospirosis, and observed higher levels of defensin α1 in AKI patients than in leptospirosis patients without AKI^23^.

### Antioxidant reduced glutathione (GSH)

GSH is an important antioxidant and low-molecular-weight peptide^38^. The kidney is highly dependent on GSH for its antioxidant properties^39^. A cohort case study conducted by Araújo *et al.* evaluated 12 patients with laboratory-confirmed leptospirosis in an emergency department and assessed oxidative stress markers and complications. In this study, GSH depletion was positively correlated with higher SCr levels; however, no significant difference was observed between leptospirosis patients with and without AKI^40^.

### Reactive oxygen species (ROS)

ROS are a group of free oxygen radicals produced during different metabolic processes that are highly reactive and potentially harmful^41^. Increased ROS levels have also been linked to CKD^42^. Araújo *et al.* included ROS in a cohort study that evaluated GSH and found that ROS levels were positively correlated with serum potassium levels. There was no significant difference between leptospirosis patients with and without AKI^40^.

### Angiopoietin-2 (Angpt-2) Asymmetric Dimethylamines (ADMA), and Symmetric dimethylarginines (SDMA)

Angpt-2, ADMA, and SDMA are markers of endothelial inflammation^43^. Angpt-2 is an important angiogenic factor that has been linked to adverse kidney outcomes in cirrhotic patients^44^ ^,^ ^45^. Dimethylarginines are guanidine metabolic residues with uremic toxicity^46^. Lukasz *et al.* conducted a retrospective cohort study using samples from patients diagnosed with leptospirosis and found higher levels of Angpt-2, ADMA, and SDMA in patients with evidence of AKI than in patients without AKI^47^.

### Osteopontin (OPN)

OPN is a highly phosphorylated glycophosphoprotein that has multiple important functions^48^. Kidneys have tubulogenic properties and other beneficial effects. Increased levels of OPN have been described in urolithiasis and kidney disease^49^. Chagan-Yasutan *et al*. reported higher levels of serum and urine OPN in patients with altered urinary dipstick parameters in a retrospective study of 112 febrile patients with confirmed acute leptospirosis^24^.

### Galectine-9 (Gal-9)

Gal-9 is a lectin from the galectin family with immunomodulatory and anti-inflammatory effects that act through the suppression of TH1 and TH17 lymphocytes^50^. In patients with type II diabetes, its levels have been linked to kidney function and the presence of CKD^51^. In a retrospective study of 112 patients with leptospirosis, Chagan-Yasutan *et al.* found an association between Gal-9 and severe leptospirosis; however, the levels were not linked to kidney damage during leptospirosis^24^.

## DISCUSSION

*Leptospirosis*-related CKD has been linked to renal fibrosis, secondary to persistent or recurrent kidney colonization by pathogenic species, or because of unresolved AKI^52^ ^,^ ^53^. Colonization causes tubulointerstitial nephritis, followed by interstitial persistence of *Leptospira*, which causes tubulointerstitial fibrosis and, if not treated, kidney failure^54^. Studies of biomarkers related to kidney damage have centered on the development of AKI during leptospirosis, with promising results suggesting their possible use as biomarkers for the early detection of kidney alterations. Because the pathogenesis of leptospiral CKD is related to renal fibrosis, biomarkers linked to this process could become potential study targets^4^. Alteration of kidney damage biomarkers precedes that of SCr and could be used to detect kidney injury before progression to CKD^55^ ^,^ ^56^. However, as opposed to leptospirosis and AKI, there are fewer studies on kidney damage biomarkers for CKD that can be used to monitor patients.

With only 60 articles, the search results revealed a generally limited knowledge base on the use of these biomarkers for leptospirosis, despite no language or year restrictions. Of these, only 11 studies met the criteria for evaluating the performance of novel biomarkers associated with kidney injury during leptospirosis. Five were cross-sectional studies with no possibility of evaluating longitudinal changes in kidney function after confirmed leptospirosis infection. Studies tended to have a small sample size, which limited the ability to identify statistically significant differences or to evaluate potentially important patient and pathogen-level factors. 

The limited evidence does support kidney injury biomarkers as a promising tool in the management of leptospirosis, potentially applicable in the diagnosis, prognosis, and follow-up of patients. NGAL and NAG were the two most studied biomarkers and provide supportive evidence as biomarkers of kidney injury. They have also been used in leptospirosis studies as a reference point to evaluate other biomarkers of AKI^22^ ^-^ ^24^. KIM-1 is a biomarker that has shown increased levels in leptospirosis patients with evidence of CKD, suspected persistence of *Leptospira,* and reduced kidney function^20^. NAG and IL-18 have shown evidence of potential use in *Leptospira*-related CKD^21^. Syndecan-1, defensin α1, GSH, Angpt-2, ADMA, and SDMA have evidence suggesting they might be useful biomarkers in leptospirosis-related AKI^22^ ^,^ ^23^ ^,^ ^40^ ^,^ ^47^.

Further evaluation of biomarkers is needed to identify their role in prognosis following leptospirosis-associated AKI, and to help prioritize patients in highly endemic regions^57^. For example, a biomarker such as KIM-1, found to have a potentially significant capability of predicting CKD in a non-leptospirosis study^21^ ^,^ ^58^, could be used to monitor the development of CKD in patients with leptospirosis. More evidence may help integrate scientific knowledge of kidney injury biomarkers into patient care applications and everyday clinical practice. More attention needs to be paid to the link between leptospirosis and CKD, particularly in highly endemic regions where persistent and recurrent infections have been described^53^, including research on host-pathogen interactions that predispose patients to chronic damage. 

The kidney disease model may also vary about which species of *Leptospira* are involved, as not all pathogenic species have the same virulence. For example, *Leptospira interrogans* is associated with aggressive episodes of leptospirosis with a higher degree of kidney impairment^59^. In contrast, *Leptospira santarosai* favors less aggressive leptospirosis, which tends to persist in the kidney^60^. These represent two different clinical models showing a biological gradient capable of developing CKD. One would follow severe infections with higher kidney impairment, and the other would develop over a longer time because of recurrent kidney insults associated with mild or asymptomatic leptospirosis^61^. These species or strain differences in pathogenesis should be considered in future studies to evaluate biomarkers.

A 2017 review by Mansour *et al.* on renal fibrosis biomarkers not related to *Leptospira* identified that *urine transforming growth factor-beta (TGF-β), blood and urine matrix metalloproteinase-2 (MMP-2),* and *MCP-1 were* associated with adverse renal outcomes based on a longitudinal follow-up of patients^32^. No studies evaluating the role of *TGF-β and MMP-2 in leptospirosis were identified in this review. Considering their potential association with renal fibrosis in CKD, future research should consider their use in leptospirosis-associated CKD.* Urine MCP-1 has been evaluated in leptospiral-related kidney damage by Yang *et al.* with mixed results^21^. This finding could also be attributed to the two-year follow-up period and small sample size, with fibrosis not having time to manifest itself.

Kidney damage biomarkers could be a potential tool for the early detection of kidney impairment, with evidence from other disease models showing promise as a tool for identifying patients who may develop AKI^62^ and CKD^63^; however, evidence is currently limited to assessing the clinical applications of these biomarkers in leptospirosis. The small number of studies included in this review shows that more research is needed to improve our understanding of injury-related biomarkers and their use in leptospirosis. That they can be measured in urine increases their potential use in rural and low-complexity primary healthcare settings with high leptospirosis endemicity.
